# A comparison of IOLMaster 500 and IOLMaster 700 in the measurement of ocular biometric parameters in cataract patients

**DOI:** 10.1038/s41598-022-16985-8

**Published:** 2022-07-27

**Authors:** Jianhong Jiang, Xiaojing Pan, Mingming Zhou, Xiaoyun Wang, Hai Zhu, Dongfang Li

**Affiliations:** 1 Department of Ophthalmology, Qingdao Eye Hospital of Shandong First Medical University, Shandong Eye Institute, Shandong Provincial Key Laboratory of Ophthalmology-State Key Laboratory Cultivation Base, Qingdao, 266000 Shandong China; 2 Department of Urology, Qingdao Municipal Hospital Affiliated to Qingdao University, 1 Jiaozhou Road, Qingdao, 266011 Shandong China

**Keywords:** Health care, Medical research

## Abstract

To compare the agreement of ocular biometric parameters measured by IOLMaster 500 and IOLMaster 700. This is a prospective study. IOLMaster 500 and IOLMaster 700 were used to measure the axial eye length (AL), corneal flat keratometry (Kf), corneal steep keratometry (Ks), mean keratometry (Km), corneal astigmatism(CA), J0, J45, anterior chamber depth (ACD) and corneal horizontal diameter (white-to-white distance, WTW) of 518 eyes (392 patients) with cataracts. Patients were enrolled unilaterally. Subgroup analyses were done according to the AL and Km. The intraclass correlation coefficient (ICC) and Bland–Altman analysis were used to evaluate the agreement. A total of 275 eyes were analyzed. The 95% confidence interval of ICC of the mean AL, Ks, Kf, Km, J0, and ACD values measured by the two instruments are indicative of excellent reliability (P < 0.001). The measurement results of WTW show good reliability (P < 0.001). The ICC of CA is of good reliability in CA < 0.5 D group (P = 0.000) and moderate reliability in the other two groups (P = 0.000). The WTW is the widest range among 95% consistency of the limit range measured by the two instruments. The results of IOLMaster 500 and IOLMaster 700 in measuring AL, keratometry, and ACD in cataract patients are of high agreement.

## Introduction

Clinically, accurate biometrics is important to obtain good visual quality after cataract surgery. Historically, the ocular biometric parameters, especially the AL value, were mainly obtained by A-scan ultrasound. However, with an ultrasound, the probe makes contact with the cornea which affects the accuracy of the results. With advances in laser technology, an optical method based on the principles of partial coherence interferometry (PCI) has been introduced. Since then, ocular biometry has entered the era of optical biometry from ultrasonic measurement. The IOLMaster is the first contactless optical coherent biometer, making no compression on the cornea and being highly safe. It is commonly used in measuring ocular biometric parameters before cataract surgery^1–3^.

The IOLMaster 500 uses PCI with a 780 nm laser diode infrared light to measure AL. Compared to coherent ultrasound biometric technology, the partial coherent interferometric biometer has better repeatability and accuracy in AL evaluation^4,5^. However, since light penetration is affected by the opacity of the refractive interstitium, measurements may not be obtained in cases of severe lens opacity, especially lens nuclear opacities and severe posterior capsule opacities^6,7^. The updated instrument, the IOLMaster 700, is a frequency swept OCT biometer that can construct a longitudinal section of the entire eye with a wavelength of 1055 nm^6^.

The IOLMaster 700 can make continuous scans from six different angles (0°, 30°, 60°, 90°, 120° and 150°) and enable visualization^8^. The IOLMaster 500 uses six spots of light to project onto the cornea in a hexagonal pattern with a diameter of about 2.5 mm, so the distance of each spot to the visual axis is about 1.3 mm. The position of each pair of reflection spots is detected and measured by the computer; the relative positions of each pair are compared to determine corneal curvature and astigmatism as a radial measurement. In the end, all of the radius are combined together into one set of measurements showing the steepest curvature and at what axis, the flattest curvature and at what axis, and the difference between the two. IOLMaster 700 can project 18 spots on three areas of the cornea and each area is distributed with 6 spots^9^. The calculation principle of corneal curvature of IOLMaster 700 is similar to IOLMaster 500. It is worth noting that PCI and swept source optical coherence tomography (SS-OCT) are not used for corneal curvature measurement.

Different me asurement principles may lead to disparate results in terms of measurement consistency. A majority of reported studies show that the agreement of the two instruments is excellent in AL, keratometry (K), and ACD measurements^6,8^. However, previous studies lack further analysis of astigmatism or exhaustive subgroup analysis. Our innovation lies in comparing all biometric parameters that can be measured with the two instruments, including AL, curvature, ACD, WTW, more subgroups, and the use of power vectors to compare astigmatism. This study aims to compare the agreement of the two instruments in measuring the ocular biometric parameters of cataract patients and provide a basis of reference for clinical applications.

## Methods

### Study design and setting

This is a prospective study. We have continuously selected patients for cataract surgery in Qingdao Eye Hospital from July to November in 2019. All subjects received slit-lamp microscopy, ophthalmoscopy, OCT, and B-scan examinations before biological examinations. Exclusion criteria were corneal diseases, glaucoma, retinal diseases and other eye diseases, as well as persons who had eye surgery, trauma, or corneal contact lens use within three months of measurement acquisition.

All measurements were taken by the same doctor who was proficient in operating the instrument using IOLMaster 500 (Carl-Zeiss company, Germany) and IOLMaster 700 (Carl-Zeiss company, Germany) biometers. During measurement, the doctor instructed patients to gaze at the gaze light in the instrument so that the measurement path and visual axis were kept consistent. Before the measurement, the doctor instructed each patient to open their eyes after blinking to form a smooth tear film on the corneal surface. Measurement results were selected from the average results with the noise-signal ratio > 10 of IOLMaster 500 and confirmed by a green signal quality indicator, which means “OK”. Additionally, an image of the fovea from the IOLMaster 700 provides another confirmation.

All measurements were performed with natural pupils to compare the correlation and consistency of the two inspection instruments in measuring AL, Kf, Ks, Km, CA, J0, J45, ACD, and WTW. The refractive index of 1.3375 was used to convert the radius of curvature (mm) into the refractive power of the cornea in diopters for both devices.

Eyes were divided into six subgroups based on AL: AL < 22.00 mm, 22.00 mm ≤ AL < 23.00 mm, 23.00 mm ≤ AL < 24.00 mm, 24.00 mm ≤ AL < 25.00 mm, 25.00 mm ≤ AL < 26.00 mm, AL ≥ 26.00 mm. Eyes were divided into three subgroups based on Km: Km < 42.00 D, 42.00 D ≤ Km ≤ 46.00 D, and Km > 46.00 D. Eyes were divided into three subgroups based on CA: CA < 0.5, 0.5 ≤ CA ≤ 0.1, and CA > 0.1.

### Statistical analysis

SPSS 17.0 and MedCalc statistical software were used for statistical methods to analyze and process the data. Mean ± standard deviation (*x* ± *S*) is often used for the expression of measurement data. The ICC and Bland–Altman were used to analyze the agreement of the measurement results from the two instruments. Intraclass correlation coefficient estimates and their 95% confidence intervals were calculated using SPSS 17.0 based on a single-rating, absolute-agreement, 2-way random-effects model. Based on the 95% confidence interval of the intraclass correlation coefficient estimate, values less than 0.5, between 0.5 and 0.75, between 0.75 and 0.9, and greater than 0.90 are indicative of poor, moderate, good, and excellent reliability, respectively^10^. The *P* < 0.05 difference is statistically significant.

### Ethical approval

The study was performed in accordance with the ethical standards as laid down in the 1964 Declaration of Helsinki and its later amendments or comparable ethical standards. Study was approved by the Institutional Review Board of Qingdao Eye Hospital of Shandong First Medical University.

### Informed consent

Informed consent was obtained from all individual participants included in the study.

### Consent for publication

Patients signed informed consent regarding publishing their data.

## Results

### Basic information

Three hundred ninety-two cataract patients who underwent ocular biometry measurements by IOLMaster 500 and IOLMaster 700 before cataract surgery were enrolled in this study. There were a total of 518 eyes used. Of them, 91 eyes (17.6%) were excluded due to failure of AL measurement by IOLMaster 500, and 36 eyes (6.9%) were excluded due to failure of AL measurement by both IOLMaster 500 and IOLMaster 700. Each patient was enrolled unilaterally, which was selected at random, so an additional 116 eyes were excluded. Finally, a total of 275 eyes of 275 patients 34–90 years old were analyzed, having an average age of 66.48 ± 10.98 years.

The mean biometric measurements with IOLMaster 500 and IOLMaster 700 are shown in Table 1.

**Table 1:** The results of biometry measurements by IOL Master 500 and IOL Master 700.

Parameter	Eyes (n)	IOLMaster 500 (mm)	IOLMaster 700 (mm)	Intraclass Correlation	95% confidence interval	P
AL (mm)	275	24.23 ± 2.39	24.24 ± 2.39	1.000	1.000–1.000	0.000
Kf (D)	275	43.92 ± 1.64	43.83 ± 1.65	0.991	0.985–0.994	0.000
Ks (D)	275	44.93 ± 1.71	44.83 ± 1.71	0.993	0.984–0.996	0.000
CA (D)	275	1.01 ± 0.66	1.00 ± 0.66	0.919	0.899–0.936	0.000
Km (D)	275	44.42 ± 1.65	44.33 ± 1.65	0.995	0.985–0.998	0.000
J0	275	− 0.15 ± 0.52	− 0.13 ± 0.51	0.953	0.940–0.963	0.000
J45	275	0.06 ± 0.26	0.03 ± 0.28	0.825	0.782–0.860	0.000
ACD (mm)	275	3.03 ± 0.47	3.00 ± 0.47	0.952	0.937–0.963	0.000
WTW (mm)	275	11.50 ± 0.43	11.60 ± 0.44	0.903	0.770–0.948	0.000

Additional subgroup analyses were done according to the AL (Table 2), Km (Table 3) and CA (Table 4) of the eyes. Four eyes counted into different AL subgroups were excluded. Five eyes counted into different Km subgroups were excluded. Seventy-six eyes counted into different CA subgroups were excluded.

**Table 2:** The subgroup analysis of AL measurement and the ICC analysis.

Groups	Eyes (n)	IOLMaster 500 (mm)	IOLMaster 700 (mm)	Intraclass correlation	95% confidence interval	P
AL < 22.00 mm	37	21.43 ± 0.98	21.43 ± 0.98	1.000	1.000–1.000	0.000
22.00 mm ≤ AL < 23.00 mm	49	22.52 ± 0.25	22.53 ± 0.26	0.995	0.988–0.997	0.000
23.00 mm ≤ AL < 24.00 mm	79	23.51 ± 0.29	23.52 ± 0.29	0.996	0.988–0.998	0.000
24.00 mm ≤ AL < 25.00 mm	31	24.35 ± 0.30	24.36 ± 0.30	0.997	0.988–0.999	0.000
25.00 mm ≤ AL < 26.00 mm	18	25.39 ± 0.33	25.40 ± 0.34	0.998	0.992–0.999	0.000
AL ≥ 26.00 mm	57	27.99 ± 1.87	27.98 ± 1.88	1.000	1.000–1.000	0.000

**Table 3:** The subgroup analysis of Km measurement and the ICC analysis.

Groups	Eyes (n)	IOLMaster 500 (D)			IOLMaster 700 (D)			Intraclass correlation		95% confidence interval		P
		Km	J0	J45	Km	J0	J45	J0	J45	J0	J45	
Km < 42.00D	13	40.89 ± 1.01	− 0.47 ± 0.51	0.16 ± 0.32	40.79 ± 0.97	− 0.42 ± 0.47	0.15 ± 0.32	0.966	0.967	0.893–0.989	0.899–0.990	0.000
42.00D ≤ Km ≤ 46.00D	218	44.11 ± 1.00	− 0.16 ± 0.49	0.05 ± 0.24	44.02 ± 1.01	− 0.13 ± 0.49	0.02 ± 0.26	0.944	0.826	0.927–0.957	0.776–0.866	0.000
Km > 46.00D	39	47.13 ± 0.81	− 0.07 ± 0.59	0.05 ± 0.30	47.07 ± 0.80	− 0.07 ± 0.57	0.04 ± 0.34	0.969	0.824	0.942–0.984	0.689–0.904	0.000

**Table 4:** The subgroup analysis of CA measurement and the ICC analysis.

Groups	Eyes (n)	IOLMaster 500 (D)			IOLMaster 700 (D)			Intraclass correlation		95% confidence interval		P
		CA	J0	J45	CA	J0	J45	J0	J45	J0	J45	
CA < 0.5D	37	0.29 ± 0.16	− 0.02 ± 0.12	0.03 ± 0.10	0.26 ± 0.11	− 0.02 ± 0.10	− 0.01 ± 0.10	0.560	0.506	0.289–0.747	0.217–0.712	0.000
0.5D ≤ CA ≤ 0.1D	77	0.74 ± 0.14	− 0.08 ± 0.30	0.06 ± 0.20	0.73 ± 0.14	− 0.05 ± 0.31	0.02 ± 0.21	0.910	0.818	0.860–0.942	0.714–0.884	0.000
CA > 0.1D	85	1.77 ± 0.60	− 0.32 ± 0.79	0.09 ± 0.36	1.75 ± 0.63	− 0.31 ± 0.78	0.09 ± 0.41	0.986	0.864	0.979–0.991	0.798–0.909	0.000

### Ocular biometric parameters and the intraclass correlation coefficient analysis

The biometric measurements measured by the two instruments and the ICC results are shown in Tables 1, 2, 3, 4, 5 and 6. Based on the 95% confidence interval of an ICC estimate, the mean AL, Ks, Kf, Km, J0, and ACD values measured by the two instruments are indicative of excellent reliability (P = 0.000). The mean CA, J45, and WTW values measured by the two instruments are indicative of good reliability (P = 0.000). In subgroup analysis, the 95% confidence intervals of an ICC in AL measurements are all above 0.988. The ICC of CA has good reliability in the CA < 0.5 D group (P = 0.000) and moderate reliability in the other two groups (P = 0.000).

**Table 5:** The results of IOLMaster 500 and IOLMaster 700 in ACD measurement and ICC analysis.

Groups	Eyes (n)	IOLMaster 500 (mm)	IOLMaster 700 (mm)	Intraclass correlation	95% confidence interval	P
AL < 22.00 mm	37	2.42 ± 0.36	2.42 ± 0.36	0.955	0.915–0.977	0.000
22.00 mm ≤ AL < 23.00 mm	49	2.83 ± 0.35	2.79 ± 0.38	0.925	0.870–0.957	0.000
23.00 mm ≤ AL < 24.00 mm	79	3.08 ± 0.38	3.06 ± 0.42	0.904	0.854–0.938	0.000
24.00 mm ≤ AL < 25.00 mm	31	3.14 ± 0.29	3.09 ± 0.34	0.894	0.791–0.948	0.000
25.00 mm ≤ AL < 26.00 mm	18	3.47 ± 0.45	3.40 ± 0.39	0.947	0.808–0.982	0.000
AL ≥ 26.00 mm	57	3.36 ± 0.30	3.30 ± 0.32	0.892	0.758–0.945	0.000

**Table 6:** The results of IOLMaster 500 and IOLMaster 700 in WTW measurement and ICC analysis.

Groups	Eyes (n)	IOLMaster 500 (mm)	IOLMaster 700 (mm)	Intraclass correlation	95% confidence interval	P
AL < 22.00 mm	37	11.14 ± 0.38	11.27 ± 0.40	0.812	0.550–0.914	0.000
22.00 mm ≤ AL < 23.00 mm	49	11.38 ± 0.35	11.44 ± 0.33	0.899	0.806–0.946	0.000
23.00 mm ≤ AL < 24.00 mm	79	11.56 ± 0.40	11.66 ± 0.42	0.912	0.759–0.958	0.000
24.00 mm ≤ AL < 25.00 mm	31	11.61 ± 0.32	11.73 ± 0.29	0.786	0.502–0.912	0.000
25.00 mm ≤ AL < 26.00 mm	18	11.83 ± 0.36	11.95 ± 0.42	0.887	0.575–0.963	0.000
AL ≥ 26.00 mm	57	11.56 ± 0.47	11.68 ± 0.46	0.909	0.751–0.958	0.000

### Bland–Altman analysis

**Figure 1:**
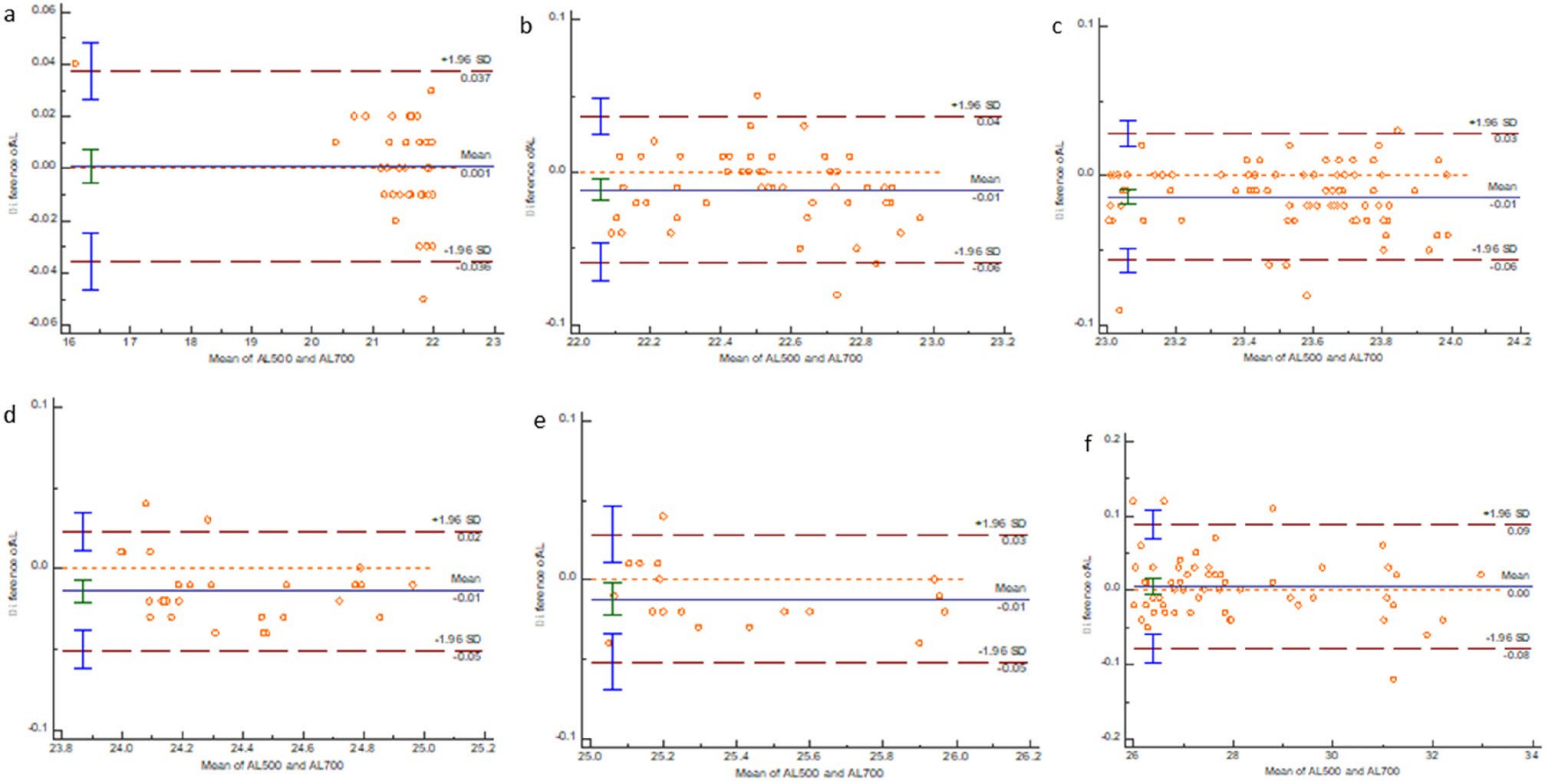
The Bland–Altman plot showing the results of AL measurement by the two instruments. (**a**) AL < 22.00 mm group. (**b**) 22.00 mm ≤ AL < 23.00 mm group. (**c**) 23.00 mm ≤ AL < 24.00 mm group. (**d**) 24.00 mm ≤ AL < 25.00 mm group. (**e**) 25.00 mm ≤ AL < 26.00 mm group. (**f**) AL ≥ 26.00 mm group.

**Figure 2:**
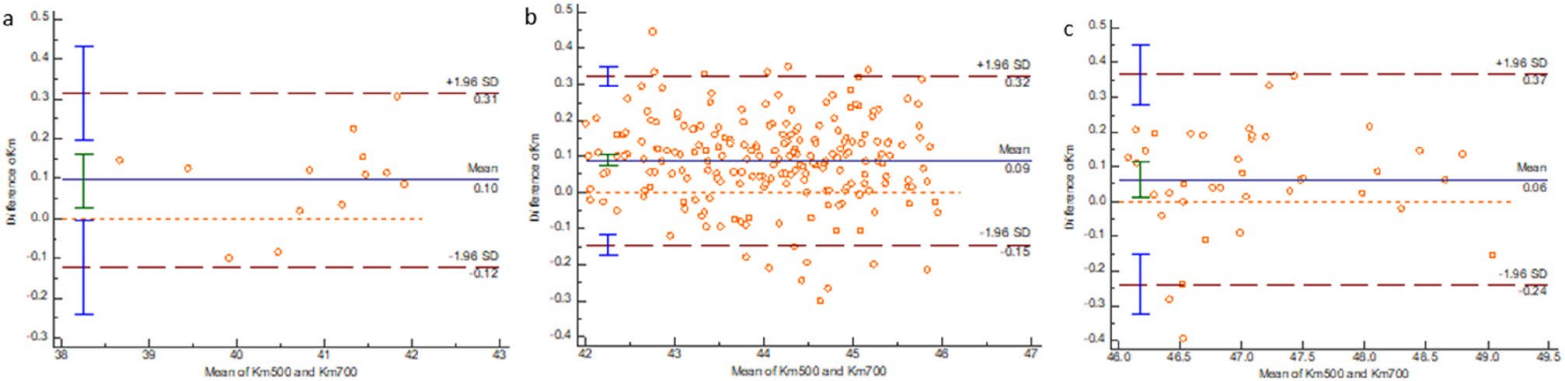
Bland–Altman plot showing the results of Km measurement by the two instruments. (**a**) Km < 42.00 D group. (**b**) 42.00 D ≤ Km ≤ 46.00 D group. (**c**) Km > 46.00 D group.

**Figure 3:**
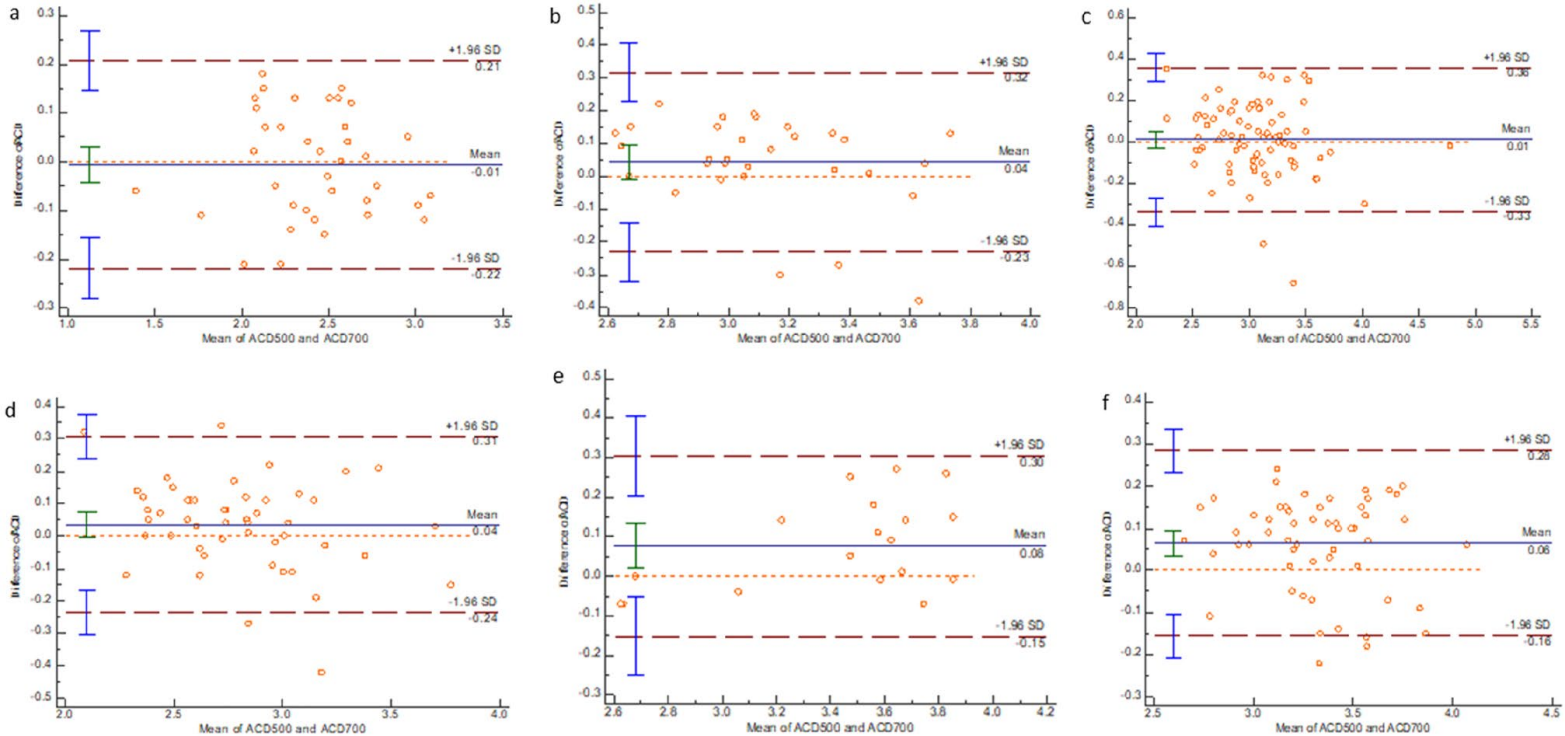
Bland–Altman plot showing the results of ACD measurement by the two instruments. (**a**) AL < 22.00 mm group. (**b**) 22.00 mm ≤ AL < 23.00 mm group. (**c**) 23.00 mm ≤ AL < 24.00 mm group. (**d**) 24.00 mm ≤ AL < 25.00 mm group. (**e**) 25.00 mm ≤ AL < 26.00 mm group. (**f**) AL ≥ 26.00 mm group.

**Figure 4:**
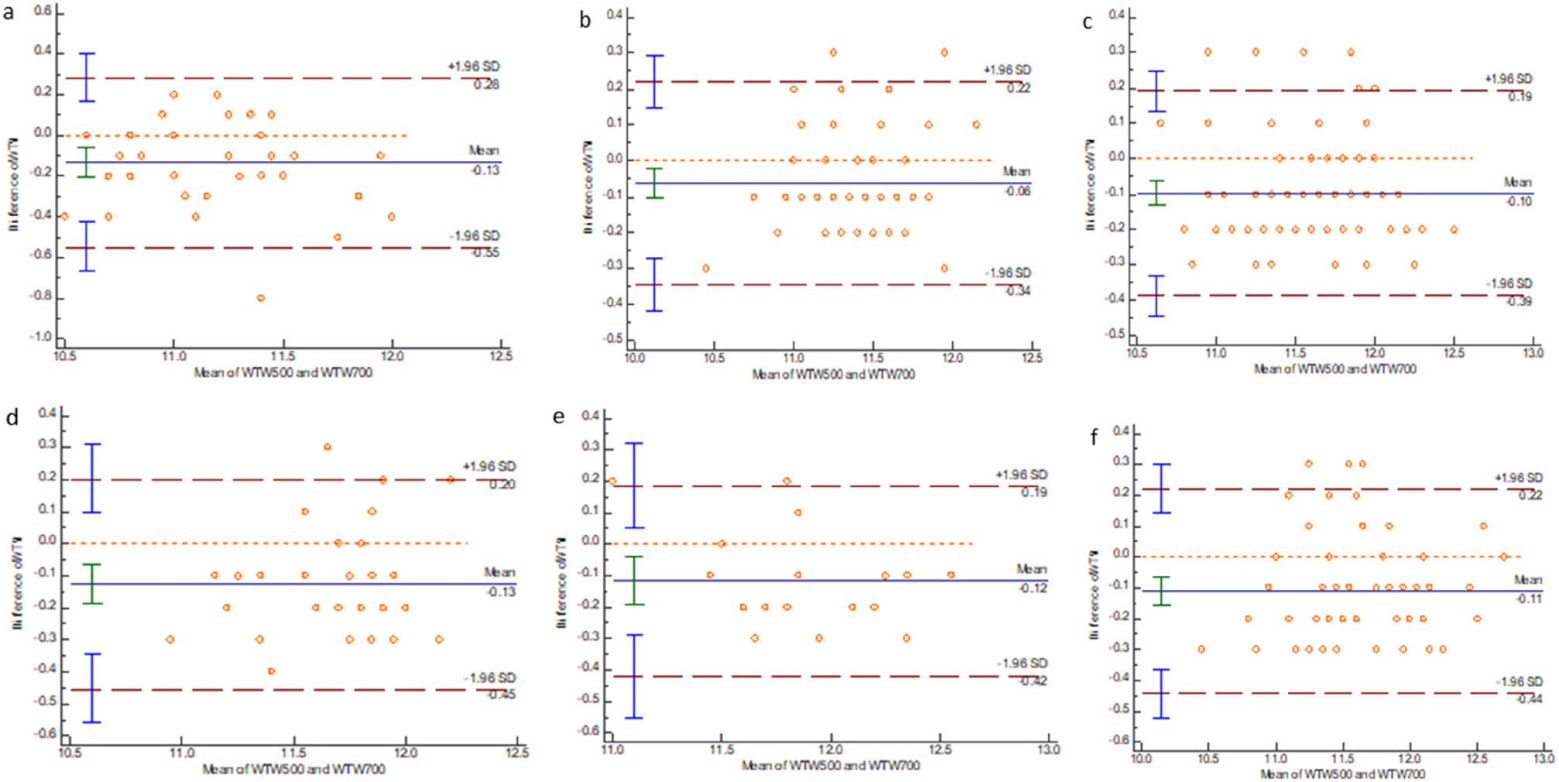
Bland–Altman plot showing the results of WTW measurement by the two instruments. (**a**) AL < 22.00 mm group. (**b**) 22.00 mm ≤ AL < 23.00 mm group. (**c**) 23.00 mm ≤ AL < 24.00 mm group. (**d**) 24.00 mm ≤ AL < 25.00 mm group. (**e**) 25.00 mm ≤ AL < 26.00 mm group. (**f**) AL ≥ 26.00 mm group.

The Bland–Altman plots of agreement between the two instruments are shown in Figs. 1, 2, 3 and 4.

## Discussion

In recent years, with the continuous improvement of cataract surgery technology, improvement of surgical equipment and the continual updates of functional intraocular lenses, doctors have gradually turned to refractive surgery to remove cataracts. Patients also have increasingly higher requirements for visual quality, which makes precise biological measurement especially important^11,12^. Our study compared the agreement of IOLMaster 500 and IOLMaster 700 in measuring the ocular biometric parameters of cataract patients.

In this study, we have found that the mean AL difference between IOLMaster 500 and IOLMaster 700 is 0.01 mm (P < 0.001). Furthermore, we showed that 95% of the confidence interval of the IOLMaster 500 and IOLMaster 700 measurement of the AL is relatively narrow, the average difference of each group is small, and the 95% limits of agreement have a small range. These findings suggest that both instruments have a relatively excellent agreement in the AL measurements results. Shi et al. also reported a good agreement between the two instruments in measuring AL values^13^. Olsen believes that among the errors that occur between the actual refractive diopter of the eyeball and the expected refractive diopter after cataract surgery, 54% come from the AL, 38% come from the prediction of the postoperative ACD, and 8% come from the evaluation of the keratometry^14–16^. Therefore, we performed a subgroup analysis of AL. The subgroup analysis revealed that the mean AL differences were 0.01 mm in AL ≥ 22.00 mm eyes and 0.00 mm in AL < 22.00 mm eyes. In the meanwhile, the ICCs of all subgroups were higher than 0.988. Thus, the AL values of the two instruments can be interchanged clinically. Akman et al.^6^ also found a small difference (0.005 mm) between the IOLMaster 500 and IOLMaster 700 and a substantial correlation, with an ICC value in AL measurements.

In the present study, 17.6% of eyes were not measured successfully with IOLMaster 500, and 6.9% were not measured successfully with IOLMaster 700. Cui et al.^17^ reported that AL measurement failed in 118 eyes (14%) using the IOL Master 500 and 55 eyes (6.5%) using the IOL Master 700. Although a slight difference may exist, our study shows the same tendencies as the previous studies. Song et al.^8^ reported that 9.7% of eyes failed with IOLMaster 500, and 2.4% of eyes with IOLMaster 700. Hirnschall et al.^18^ reported a 6.4% and 0.5% failure rate, respectively. We have a higher failure rate, as a result of some patients’ reluctance to have medical treatment in the early stage of cataract.

Keratometry has an important guiding significance for measuring intraocular lens diopter of patients receiving age-related cataract surgery, the selection of surgical incisions, and the correction of astigmatism^19^. In the measurement of Km, our study shows that IOLMaster 500 measured a steeper Km value compared to IOLMaster 700. It was consistent with a study by JS Song^8^. Both IOLMaster 500 and IOLMaster 700 measure the cornea’s curvature by projecting a light source onto the cornea^9^. However, the different measurement methods cause a slight difference in keratometry results. Due to the importance of astigmatism meridian and astigmatism magnitude, both J0 and J45, which are believed more appropriate for astigmatism measurements, were also included in the evaluation of astigmatisms^20,21^. We found that these two instruments have good consistency in measuring J0 and J45. However, subgroup analysis revealed that the low astigmatism group was of good to moderate reliability. It has been considered that a lower astigmatism value is associated with bigger differences^22^. The ICC was lower for J45 than for J0. We considered that since the two instruments have more points coincident in the horizontal and vertical directions, J45 would be relatively less consistent than J0.

For ACD measurements, we found a significant difference between the instruments—the IOLMaster 500 measured a longer ACD than IOLMaster 700. Similar differences were found by Akman et al.^6^, while Srivannaboon et al.^23^ reported that the IOLMaster 700 measured a longer ACD than the IOL Master 500 but with no significant difference. The principle of the IOLMaster 500 for measuring ACD is based on an optical section through the anterior chamber using a lateral slit beam illumination technique, while the IOLMaster 700 detects the longitudinal section of the eye by SS-OCT to measure the ACD^8^. Differences in these measurement principles would have resulted in differences in the ACD measurement. However, based on the 95% confidence interval of an ICC estimate, ACD measured by the two instruments is of excellent reliability.

For both instruments, WTW is measured by a light-emitting diode light source to detect the edge image of the iris. In the present study, we found that IOLMaster 500 and IOLMaster 700 had a good agreement in measuring WTW, except for AL values less than 22.00 mm and AL values ranging from 24.00 to 26.00 mm, which had a moderate agreement. Shi et al. reported that the difference between the two instruments was statistically significant; nevertheless, the ICC and Bland–Altman plot analysis indicated that the consistency of the two instruments for the WTW measurement was good^13^. The difference found in this study concerning the results in the WTW measurement is not clinically important in practical application. Besides, we found that ACD and WTW increased as the AL increased, except for AL ≥ 26.0 mm. It is consistent with the characteristics seen in a previous study^24^. WTW is included in the calculation of the fourth generation IOL power calculation formulas, including Holladay 2, Olsen, Hill-RBF, and Barrett Universal II^25^. It has been reported that WTW is significantly related to ACD and is affected by the equivalent spherical lens power of the patient^26^. WTW has a very small effect on IOL power calculation.

In summary, the measurement results of IOLMaster 500 and IOLMaster 700 are outstandingly agreeable in terms of axial eye length, keratometry and central anterior chamber depth measurements of cataract patients. Inevitably, there are shortcomings in this study. For example, the tracking of intraocular lens diopter and postoperative vision and the measurement of the intraocular lens are lacking. The degrees of cataract are not classified, which may affect the measurement results. Further studies are needed concerning the feasibility and consistency of IOLMaster 500 and IOLMaster 700 in terms of their biometric measurement of the eyeball in complex patients after refractive surgery or fundus disease.

## Data Availability

Data are available at the authors.
